# Evolocumab in the treatment of refractory chylothorax induced by systemic lupus erythematosus complicated with hyperlipidemia: a case report and literature review

**DOI:** 10.3389/fimmu.2026.1819213

**Published:** 2026-08-13

**Authors:** XiuShi Li, AnMao Li, Mei Tian, XiuShan Chen, HaoQi Zhao, ZhuoYan Dai, JunYi Li, ChunYan Li

**Affiliations:** 1 Cardiology Department, Affiliated Hospital of Zunyi Medical University, Zunyi, Guizhou, China; 2 Department of Rheumatology and Immunology, Affiliated Hospital of Zunyi Medical University, Zunyi, Guizhou, China; 3 Clinical College, Zunyi Medical University, Zunyi, Guizhou, China

**Keywords:** chylothorax, evolocumab, hyperlipidemia, pleural effusion, systemic lupus erythematosus

## Abstract

Recurrent chylothorax induced by thoracic duct injury in patients complicated with mixed hyperlipidemia secondary to systemic lupus erythematosus (SLE) is rarely encountered in clinical practice. A 55-year-old female patient diagnosed with SLE, whose predominant clinical manifestation was refractory pleural effusion, is reported in this paper. Combined immunosuppressive therapy consisting of glucocorticoids, cyclophosphamide (CYP) and hydroxychloroquine (HCQ) was administered to the patient for 8 months. Although the disease activity of SLE was effectively controlled, intermittent albumin infusion, closed thoracic drainage and intensive lipid-lowering therapy were supplemented during the treatment period, yet recurrent pleural effusions were still detected. At this stage, low-density lipoprotein (LDL) was reduced below the target level, while total cholesterol (TC) and triglyceride (TG) were persistently maintained at elevated levels. Chylous pleural effusion was confirmed by pleural fluid examinations. Thoracic duct injury was presumed to be triggered by two combined factors including active SLE and hyperlipidemia. Intensive lipid-lowering treatment was therefore required on the basis of effective control of SLE activity. Evolocumab, a representative drug of proprotein convertase subtilisin/kexin type 9 inhibitors (PCSK9i), was selected to further strengthen blood lipid regulation. Serum lipid levels were restored to normal ranges during the treatment course. Pleural effusions were effectively controlled without recurrence, and serum albumin levels were gradually normalized. Positive therapeutic effects of PCSK9 inhibitors on patients suffering from refractory chylothorax caused by SLE combined with mixed hyperlipidemia are indicated by this case.

## Introduction

Systemic Lupus Erythematosus (SLE) is a complex autoimmune disease characterized by multi-organ involvement and the presence of various autoantibodies in the serum. This disease is closely related to immune complex deposition, complement deficiencies, and immune cell dysfunction (1). Pleural effusion is a common manifestation of lupus pleuritis (2). Unlike serous or hemorrhagic pleural effusions, chylothorax is characterized by elevated chylomicrons and triglycerides and is a rare complication of SLE, usually associated with immune activity and lymphatic and thoracic duct inflammation caused by dyslipidemia (3–6). Although most cases can be alleviated through conventional glucocorticoid combined with immunosuppressive therapy or surgical intervention, a standardized treatment protocol for SLE-related chylothorax has yet to be established (7, 8). Intensive blood lipid regulation can be achieved with evolocumab, which is a representative agent of proprotein convertase subtilisin/kexin type 9 inhibitors (PCSK9i), and favorable therapeutic effects can be exerted on patients with intractable dyslipidemia (9). A case of refractory chylothorax secondary to systemic lupus erythematosus complicated with hyperlipidemia that was successfully treated with PCSK9i is reported in the present study, and the potential clinical value of PCSK9i with dual lipid-regulating and anti-inflammatory effects is discussed.

## Case report

**Figure 1:**
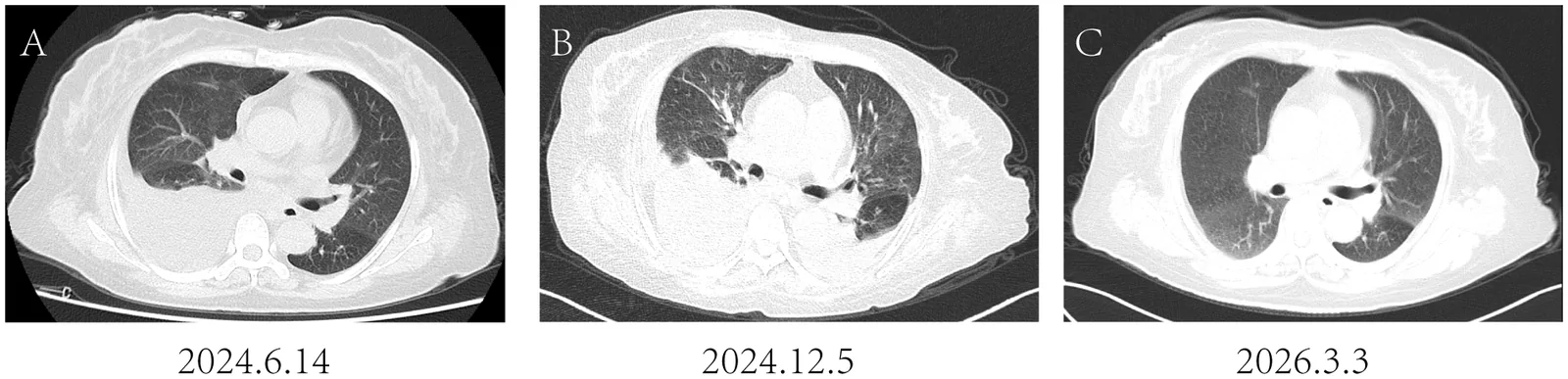
Serial chest CT images of a patient: **(A)** Before treatment; **(B)** After glucocorticoid and immunosuppressive therapy; **(C)** After PCSK9 inhibitor therapy CT, computed tomography, PCSK9, proprotein convertase subtilisin/kexin type 9.

A 55-year-old female patient was admitted to the hospital in June 2024 due to bilateral lower limb edema accompanied by shortness of breath. During the course of the illness, she experienced bilateral lower limb edema with dyspnea on exertion and was unable to lie flat at night. There was no hair loss, photosensitivity, oral or genital ulcers, fever, or cough. Urine output was approximately 1000ml/day, with no foamy urine. The patient’s past medical history was unremarkable, with no prior hospitalizations or surgical interventions. She took no regular medications. Her family history was noncontributory. Physical examination on admission revealed a temperature of 36.5 °C, heart rate 78 beats/min, respiratory rate 25 breaths/min, and blood pressure 132/80 mmHg. Pulmonary auscultation demonstrated diminished breath sounds with dullness to percussion over the right lower chest. No cardiac murmurs, pericardial friction rub, or abdominal abnormalities were detected. Moderate, symmetric pitting edema was noted in both lower extremities. Imaging examinations revealed a large right-sided pleural effusion and a small amount of pericardial effusion (**Figure 1A**). Antinuclear antibody(ANA)1:1280, with positive anti-SSA and anti-SSB antibodies (+++) and anti-Sm antibodies (++), and complement levels were significantly reduced (C3 0.54 g/L, C4 0.16 g/L). The erythrocyte sedimentation rate (ESR) was significantly elevated (120 mm/hour). The Schirmer test was <5mm/5min. The three antiphospholipid antibodies, lupus anticoagulant ratio, and anti-β2 glycoprotein antibodies were all normal, and multiple urine protein tests were negative. Based on the 2019 EULAR/ACR SLE and 2016 EULAR/ACR primary Sjögren’s syndrome (PSS) classification criteria, the patient was diagnosed with SLE complicated with Sjögren’s syndrome. Key negative results included (pANCA, MPO, cANCA, PR3), effectively ruling out vasculitis. Routine analysis of the pleural effusion showed a pale yellow appearance, weakly positive Rivalta’s test, a total cell count of 606 × 10^6^/L, a total nucleated cell count of 532 × 10^6^/L, with 25% polymorphonuclear cells and 75% mononuclear cells. Pleural effusion biochemistry revealed a total protein of 14.0 g/L, glucose of 11.6 mmol/L, lactate dehydrogenase of 73 U/L, and adenosine deaminase of 5.40 U/L. According to Light’s criteria (protein/serum protein > 0.5, LDH/serum LDH > 0.6), it was determined to be an exudate. Although carbohydrate antigen 19-9 (CA-199) levels were elevated, subsequent gastrointestinal endoscopy did not reveal malignancy, and other tumor markers were negative, ruling out malignancy.

**Figure 2:**
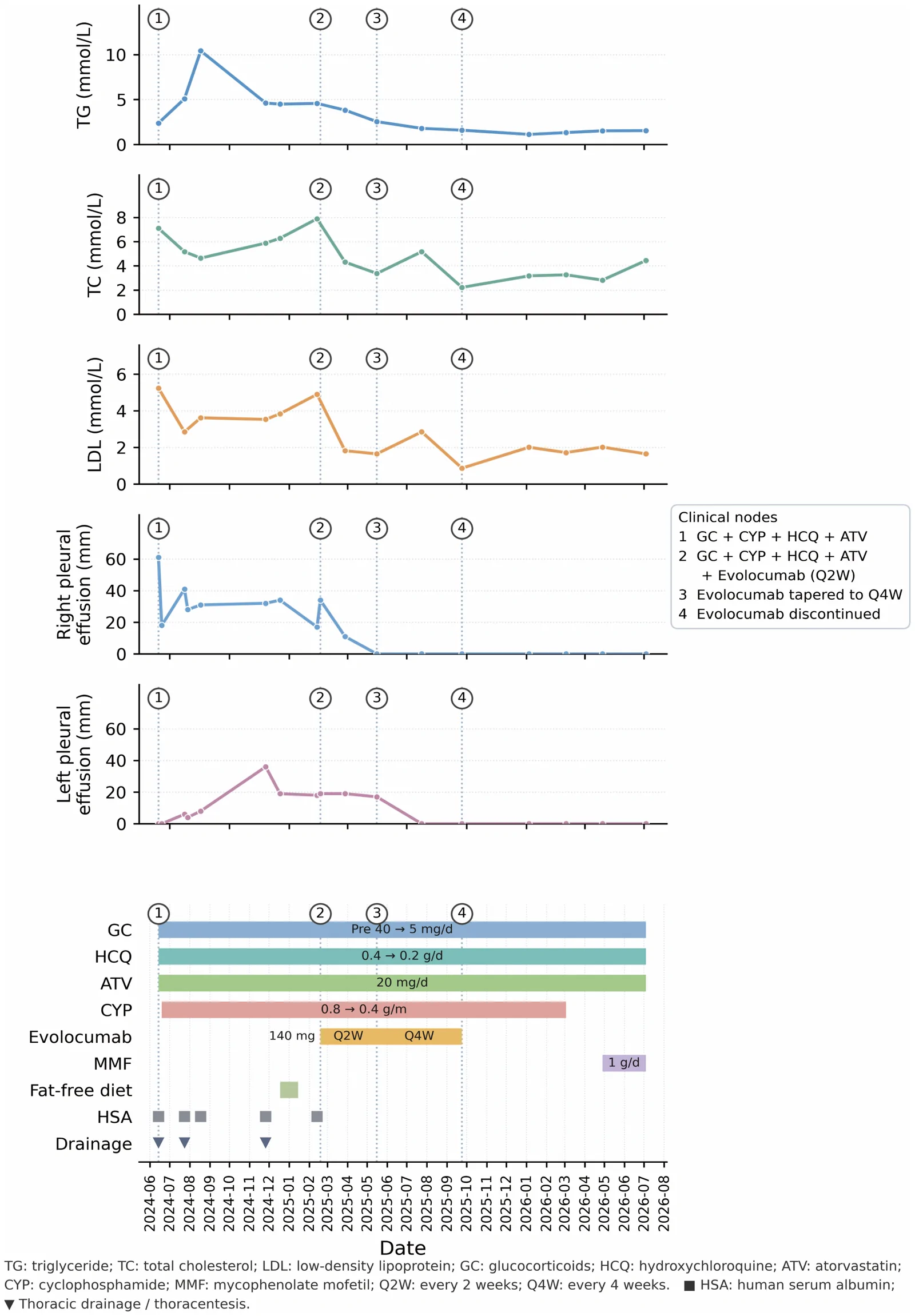
Clinical course, treatment interventions, lipid levels and color Doppler ultrasound pleural effusion depth over time. The upper panels depict the longitudinal trajectories of triglyceride (TG), total cholesterol (TC), low-density lipoprotein (LDL), and bilateral pleural effusion depths throughout the follow-up period. The lower timeline provides a chronological summary of the primary therapeutic interventions and major clinical milestones. After Evolocumab initiation, serum lipid levels returned to normal and pleural effusion showed gradual remission, both of which were maintained within the physiological ranges during the remainder of follow-up.

The patient was also found to have hypercholesterolemia, hypertriglyceridemia, and hypoproteinemia. To control the large pleural effusion and dyslipidemia, she received intermittent albumin infusions and multiple closed chest drainage procedures, along with atorvastatin 20mg orally once daily to manage blood lipids and a standard immunosuppressive treatment regimen for SLE, including an initial dose of methylprednisolone 40mg once daily for six days, followed by a switch to prednisone 40mg/day orally after discharge, intravenous CYP (0.8g/month), and HCQ (400mg/day). After 8 months of follow-up, the prednisone was reduced to 10mg/day for maintenance, and CYP accumulated to 7.2g. A score of 2 points (secondary to pleuritis) was assigned according to the SLE Disease Activity Index 2000 (SLEDAI-2K), and mild disease activity was identified; nevertheless, the pleural effusion failed to be effectively controlled (**Figure 1B**). Further chylous tests of pleural fluid were completed, and positive results were acquired. The triglyceride level in pleural fluid was measured higher than 110 mg/dL, and the cholesterol/triglyceride ratio was determined less than 1, through which chylous pleural effusion was diagnosed. A diagnostic challenge was the unavailability of thoracic duct angiography—the gold standard for localizing chylous leakage—at our institution. Consequently, the exact site of thoracic duct injury could not be confirmed, and the diagnosis relied on clinical presentation, pleural fluid biochemical analysis, and exclusion of alternative etiologies. Thoracic duct magnetic resonance imaging (MRI) was performed, and no abnormal manifestations were detected. Consultations with thoracic surgery specialists were conducted, and unilateral chylothorax was considered to be induced by thoracic duct injury resulting from mixed hyperlipidemia. No history of thoracic surgery or trauma was reported by the patient after inquiry. No signs of tuberculosis or malignancy were presented on thoracic computed tomography (CT). Repeated routine, biochemical, bacterial, tuberculous and parasitic examinations of pleural fluid were all normal, and pleural effusions induced by concurrent infection or tuberculosis were excluded. Strict dietary fat restriction was recommended by specialists, including fat-free diet, medium-chain triglyceride (MCT) oil and high-quality protein diet. No improvements were observed after more than four weeks of strict dietary intervention, which indicated that limited efficacy could be achieved by dietary regulation alone. Given that thoracic duct injury was presumed to be jointly induced by two factors including active SLE and mixed hyperlipidemia, an intensive lipid-lowering regimen was initiated on the basis of effective control of SLE disease activity, and combined treatment with atorvastatin and evolocumab was administered to the patient. A total follow-up period of approximately 16 months was conducted after the initiation of evolocumab administration. Subcutaneous injection of 140 mg evolocumab once every two weeks was delivered within the initial three months, and serum lipid levels were restored to normal ranges during this follow-up phase.The administration scheme was then adjusted to 140 mg evolocumab injected subcutaneously once every four weeks for four months of maintenance therapy. Complete absorption of pleural effusion was demonstrated by re-examined thoracic imaging after combination therapy (**Figure 1C**). Nine months of continuous follow-up was implemented after drug withdrawal. The SLEDAI-2K score was re-evaluated as 0 point, complete remission of SLE was confirmed, normal lipid levels were sustained, and no recurrence of pleural effusion was observed. The overall therapeutic schedule and dynamic changes of laboratory indicators are presented in **Table 1**, and the temporal variation trends of serum lipid levels and pleural effusion depth measured by color Doppler ultrasound under different lipid-lowering regimens are illustrated in **Figure 2**. Medication dispensing records and injection archives were reviewed, and strict compliance with medical orders was verified in this patient. No adverse reactions were documented during the whole medication period, and good drug tolerance was exhibited by the patient. The patient reported marked improvement in dyspnea within two months of evolocumab initiation. She regained the ability to perform daily activities without shortness of breath and resumed supine sleeping. She expressed satisfaction with the outcome and relief at avoiding surgical intervention. No injection-related discomfort or other adverse events were documented throughout the treatment course. Informed written consent was obtained from the patient for publication of this report and any accompanying images.

**Table 1:** Laboratory results and therapeutic course.

Date	TG	TC	LDL	HSA	CYP	MMF	HCQ	ATV	Right thoracic drainage	Thoracentesis	Fat-freediet	Evolocumab	GC	Depth of right pleura leffusion	Depth of left pleural effusion
	<1.7mmol/L	<5.2mmol/L	<3.4mmol/L											mm	mm
2024/6/14	2.38	7.1	5.24	90g	NO	NO	0.4g/d	20mg/d	NO	700ml	NO	NO	IP: Mp 40 mg qd	61	0
2024/6/19	–	–	–	NO	0.8g	NO	0.4g/d	20mg/d	NO	NO	NO	NO	DC: Pre 40mg qd	18	0
2024/7/24	5.09	5.17	2.85	100g	NO	NO	0.4g/d	20mg/d	YES (total drainage: 3000ml)	NO	NO	NO	IP: Mp 40 mg qd	41	6
2024/7/29	–	–	–	NO	0.8g	NO	0.4g/d	20mg/d	NO	NO	NO	NO	DC: Pre 40mg qd	28	4
2024/8/18	10.42	4.64	3.62	80g	0.8g	NO	0.4g/d	20mg/d	NO	NO	NO	NO	IP: Mp 40 mg qd	31	8
2024/9/27	–	–	–	NO	0.8g	NO	0.4g/d	20mg/d	NO	NO	NO	NO	OP: Pre 30mg qd	–	–
2024/11/1	–	–	–	NO	0.8g	NO	0.4g/d	20mg/d	NO	NO	NO	NO	OP: Pre 15mg qd	–	–
2024/11/26	4.61	5.88	3.53	220g	NO	NO	0.4g/d	20mg/d	YES (total drainage: 1400ml)	NO	NO	NO	IP: Mp 80 mg qd	32	36
2024/12/18	4.49	6.28	3.84	NO	0.8g	NO	0.4g/d	20mg/d	NO	NO	YES	NO	DC: Pre 20mg qd	34	19
2025/1/15	–	–	–	NO	0.8g	NO	0.4g/d	20mg/d	NO	NO	YES	NO	OP: Pre 15mg qd	–	–
2025/2/13	4.57	7.89	4.9	60g	0.8g	NO	0.4g/d	20mg/d	NO	NO	NO	NO	IP: Mp 20mg qd	17	18
2025/2/18	–	–	–	NO	0.8g	NO	0.4g/d	20mg/d	NO	NO	NO	YES (140mg Q2W)	DC: Pre 10mg qd	34	19
2025/3/28	3.82	4.31	1.82	NO	0.8g	NO	0.4g/d	20mg/d	NO	NO	NO	YES (140mg Q2W)	OP: Pre 10mg qd	11	19
2025/5/16	2.54	3.37	1.65	NO	0.8g	NO	0.4g/d	20mg/d	NO	NO	NO	YES (140mg Q4W)	OP: Pre 10mg qd	0	17
2025/7/24	1.79	5.17	2.85	NO	0.8g	NO	0.4g/d	20mg/d	NO	NO	NO	YES (140mg Q4W)	OP: Pre 5mg qd	0	0
2025/9/24	1.59	2.22	0.87	NO	0.4g	NO	0.4g/d	20mg/d	NO	NO	NO	YES (140mg Q4W)	OP: Pre 5mg qd	0	0
2026/1/5	1.12	3.17	2.01	NO	0.4g	NO	0.2g/d	20mg/d	NO	NO	NO	NO	OP: Pre 5mg qd	0	0
2026/3/3	1.32	3.26	1.72	NO	0.4g	NO	0.2g/d	20mg/d	NO	NO	NO	NO	OP: Pre 5mg qd	0	0
2026/4/28	1.52	2.82	2.02	NO	NO	1g/d	0.2g/d	20mg/d	NO	NO	NO	NO	OP: Pre 5mg qd	0	0
2026/7/4	1.54	4.44	1.65	NO	NO	1g/d	0.2g/d	20mg/d	NO	NO	NO	NO	OP: Pre 5mg qd	0	0

TG, triglyceride; TC, total cholesterol; LDL, low-density lipoprotein; HSA, human serum albumin; CYP, cyclophosphamide; MMF, mycophenolate mofetil capsulies; HCQ, hydroxychloroquine; ATV, atorvastatin; GC, glucocorticoids; Pre,prednisone; MP, methylprednisolone; No, not used; IP, inpatient; OP, outpatient; DC, discha; Note: pleural effusion depth was measured by color Doppler ultrasound as the maximum depth at a fixed site with the patient in a consistent position.

## Discussion

In recent years, the crosstalk between lipid metabolism and the immune system (immunometabolism) has been regarded as a cutting-edge research field for autoimmune diseases. Cholesterol metabolism is regulated by PCSK9i via the degradation of low-density lipoprotein receptors (LDLRs), and evolocumab, a representative inhibitor of this class, has been established as a first-line therapeutic agent for atherosclerotic cardiovascular diseases (9). Notably, accumulating evidence has been documented to demonstrate that the biological functions of PCSK9 extend far beyond lipid metabolism, and direct participation in the regulation of immune inflammation is mediated by PCSK9.

Direct pro-inflammatory activity is possessed by PCSK9 itself; inflammatory responses are participated in, autophagy and cell apoptosis are regulated, and immune cell functions are affected via LDLR-independent pathways, by which autoimmune reactions are aggravated. In oncology research, elevated intratumoral infiltration of CD8+ T cells has been verified after the administration of PCSK9 inhibitors, which provides a direct indication that LDLR-independent regulatory effects on T cells are exerted by PCSK9 (10). The activation of dendritic cells (DCs) and T cells induced by oxidized low-density lipoprotein (OxLDL) is markedly amplified in patients with SLE, and the maturation of DCs is PCSK9-dependent. OxLDL-triggered DC maturation is abolished after PCSK9 silencing, and one potential mechanism through which the progression of cardiovascular complications in SLE patients may be influenced by PCSK9 is highlighted by this study (11). Positive correlations between circulating PCSK9 concentrations and SLE disease activity have been identified in multiple Mendelian randomization analyses and clinical observations, and genetic causal relationships between loss-of-function variants of the PCSK9 gene and reduced susceptibility to SLE have been established (12, 13). The differentiation of naive CD4+ T cells into Th1 and Th17 subsets is promoted by PCSK9, and the secretion of pro-inflammatory cytokines is upregulated to trigger vascular inflammation (14). In patients with ankylosing spondylitis, positive correlations of serum PCSK9 levels with disease activity and the proportion of Th17 cells among CD4+ T lymphocytes have been reported by Cai et al. (15). In rheumatoid arthritis, elevated proportions of Th17 cells and increased Th17/Treg ratios are positively associated with PCSK9 expression (16). PCSK9 has been demonstrated by the above-mentioned studies to be one of the vital molecules linking metabolic disorders and autoimmune diseases, and potential therapeutic significance for autoimmune diseases can be exerted by PCSK9 inhibition.

In this case, after 8 months of GC combined with CYP treatment, although the systemic disease activity was effectively controlled, there was no reduction in the chylothorax. To further explore such refractory cases, we searched the PubMed database for case reports, case series, original studies, and English-language reviews published from January 1, 2000, to July 1, 2026, using keywords including “SLE”, “pleural effusion”, “chylothorax”, and related treatment protocols. All included literature was accessible in full text. The characteristics of the cases are summarized in **Table 2**. Ten case reports concerning patients with systemic lupus erythematosus (SLE) accompanied by chylothorax have been collected that are analogous to the present research. Conventional immunosuppressive regimens were demonstrated to be ineffective in nine of these cases, whereas clinical improvements were observed in one patient after the treatment was replaced with combined sirolimus and cyclophosphamide (CYP). Abnormal manifestations on lymphangiography were identified in five patients who showed no response to immunosuppressive therapy. Satisfactory clinical outcomes were attained in four of the five patients after surgical intervention, and concurrent parasitic infection was confirmed in the remaining single case (17–23). Unlike previous cases, this patient had persistent dyslipidemia, while the MRI of the thoracic duct did not show obstruction. This characteristic prompted us to adjust the treatment strategy, hypothesizing that directly targeting lipid metabolism might be significant for repairing thoracic duct injury and alleviating chylous effusion. Therefore, we added a PCSK9 inhibitor to the original regimen, aiming to promote disease remission through new pathways for regulating lipid metabolism. This intervention effectively reduced the patient’s low-density lipoprotein, total cholesterol, and triglyceride levels, facilitating the absorption of pleural effusion.

**Table 2:** Clinical characteristics and treatment responses in 10 patients with SLE and chylothorax.

Patient	1^1^	2^2^	3^3^	4^3^	5^3^	6^3^	7^4^	8^5^	9^6^	10^7^
Reference	Lin, 2007 (17)	Manzella, 2013 (18)	Song, 2019 (19)	Song, 2019 (19)	Song, 2019 (19)	Song, 2019 (19)	Banic, 2023 (20)	David, 2023 (21)	Kuwahara, 2024 (22)	Maryani, 2026 (23)
Age, sex	43, F	36, F	22, F	33, F	29, F	28, M	24, F	23, M	23, F	21, F
Resp. symptoms	dry cough	abdominal distension	chest tightness, dyspnea	chest tightness, dyspnea	chest tightness, dyspnea	chest tightness, dyspnea	Dyspnea	productive cough	Chronic dyspnea	Shortness of breath
Type and site of effusion	Ex, Lt	Ex, Rt	Ex, Lt	Ex	Ex	Ex	Ex, Rt	Ex, Rt	Ex	Ex
Chylothorax	Yes	Yes	Yes	Yes	Yes	Yes	Yes	Yes	Yes	Yes
Lipid	NR	Normal	NR	NR	NR	NR	NR	NR	NR	NR
Thoracic duct abnormality	NR	NR	Yes (lymphangiography: poor backflow at terminal)	Yes (lymphangiography: poor backflow)	Yes (complete obstruction)	Yes (complete obstruction)	No	NR	NR	Yes (filariasis-induced lymphatic obstruction by Brugia malayi)
Surgical treatment	No	No	Yes (surgery to release adhesion)	Yes (surgery)	Yes (thoracic duct expansion)	Yes (thoracic duct expansion)	No	No	Yes(Pleuro-peritoneal shunt + Peritoneal-venous shunt + CART)	Yes(Thoracotomy with bilateral chest tube drainage)
Lipid-lowering therapy	NR	No	NR	NR	NR	NR	NR	NR	NR	No
ESR	NR	17	75	140	NR	NR	NR	129	NR	NR
C3, C4	NR	Low C4	Low C3	Normal	Normal	Normal	Low C3	Low C3, C4	NR	NR
anti-dsDNA	+	+	+	+	+	+	–	+	NR	NR
Previous therapy and outcome	P, HCQ—for a few days cleared rapidly	P—for 2 months improvement but not elimination	P, CYP — no reduction in effusion	P, CsA — no reduction	P, CsA — no reduction	P, CYP + MTX + tamoxifen — no reduction	P, chloroquine, CYP for 2 years—no effect, pleural effusions worsened; Further add MMF—no improved	P, CYP—improve modestly	Prednisolone + Tacrolimus + Cyclophosphamide → no benefit, progressive effusion	Corticosteroids → partial
Current therapy	AZA	P, AZA for 6 months	Surgery (adhesion release)	Surgery (adhesion release)	Surgery (thoracic duct expansion)	Surgery (thoracic duct expansion)	Sirolimus 2 mg per day, low-dose P, MMF for 12 months	P, AZA	Pleuro-peritoneal shunt + Peritoneal-venous shunt + CART	DEC + Albendazole + MCT diet
Outcome	No eff. in 4 years	No eff.	Significant decrease post-surgery; remission at 10 months	Significant decrease; remission	Significant decrease; remission	Significant decrease; remission	pleural effusion volume and lung function improved, No eff. in 2 years	No eff. in 18 months	Effective for dyspnea improvement while maintaining nutrition;	Day 5: drainage 600→100 mL/24h; SOB resolved; O_2_ 88%→99%

SLE, systemic lupus erythematosus; NR, not reported; Ex, exudate; Lt, left side; Rt, right side; ESR, erythrocyte sedimentation rate; C3, complement 3; C4, complement 4; P, prednisone; HCQ, hydroxychloroquine; CYP, cyclophosphamide; CsA, ciclosporin; MMF, mycophenolate mofetil; AZA, azathioprine; DEC, diethylcarbamazine; MTC, medium-Chain Triglycerides Diet; rec, recurrence; eff, effusion.

The thoracic duct, as the largest lymphatic vessel in the human body, typically originates from the cisterna chyli located at the level of T12 to L2 vertebrae, enters the thoracic cavity through the aortic hiatus, ascends along the right anterior side of the spine, and ultimately drains into the left brachiocephalic vein. Approximately 75% of the body’s lymphatic fluid drains into the venous system via the thoracic duct. The course of the thoracic duct determines whether damage to this structure will lead to left- or right-sided chylothorax: injuries above the thoracic plane usually result in left chylothorax, while injuries below the thoracic plane typically lead to right-sided chylothorax; when damage occurs at the thoracic plane, bilateral chylothorax may occur (24). There are two main mechanisms for thoracic duct injury and chylous leakage: 1) direct trauma or rupture of the lymphatic vessel; 2) obstruction of the thoracic duct with leakage from fragile collateral vessels. Traumatic injury is the more common cause, while benign obstruction is associated with systemic diseases, infections, and other factors (25). The exact pathophysiological mechanism of SLE-related chylothorax has not been fully elucidated, but it is currently believed to be a multifactorial process involving immune inflammation, lymphatic structural damage, and hemodynamic changes (26). The typical pathological feature of SLE is the deposition of immune complexes in the vessel walls, activating complement and triggering a series of inflammatory cascade reactions. This systemic inflammation not only increases capillary permeability but also affects the lymphatic system, inducing lymphangitis. Prolonged lymphangitis can lead to narrowing or obstruction of the lumen, resulting in increased intraluminal pressure, increased wall permeability, and ultimately the formation of chylous effusion. Hyperlipidemia, as a common complication or treatment-related metabolic abnormality in SLE, plays a synergistic role in the above pathological process. Elevated lipid levels further increase the chylomicron load in lymphatic fluid, exacerbating thoracic duct pressure and promoting chylous leakage. Additionally, high concentrations of lipids and their oxidative metabolites can directly induce damage to lymphatic endothelial cells or amplify local inflammation by promoting oxidative stress responses, leading to the destruction of lymphatic wall integrity and increased fragility. The laboratory diagnosis of chylothorax primarily relies on biochemical analysis of pleural fluid. The most widely used diagnostic criterion is a pleural effusion triglyceride level > 1.24 mmol/L (110 mg/dL). Based on biochemical confirmation, imaging examinations are crucial for clarifying the site of thoracic duct injury. Enhanced chest CT can assess mediastinal structures and pleural conditions; magnetic resonance lymphangiography can non-invasively display lymphatic vessel morphology and leakage areas; while lymphangiography is the “gold standard” for locating thoracic duct damage or obstruction, serving as a key examination to identify the leakage point of the thoracic duct (5).

The therapeutic regimen of PCSK9 inhibitors was initiated based on two considerations, namely the correction of dyslipidemia and the potential alleviation of inflammatory responses. Although a 70-year-old patient was documented in a previous study to develop moderate pericardial effusion, bilateral pleural effusion and lymphadenopathy after the administration of PCSK9 inhibitors, the effusions were resolved after the discontinuation of evolocumab and the delivery of combined treatment with colchicine and prednisone (27). Such adverse reactions are regarded as extremely rare given the extensive clinical application of PCSK9 inhibitors. The adverse reactions described in that literature are deemed immune-mediated serositis induced by monoclonal antibodies, and the activation of aberrant immune and inflammatory reactions is identified as its core mechanism. Nevertheless, the pathophysiological mechanism of the chylous pleural effusion reported in the present study is completely distinct. Thoracic duct injury and lymphatic leakage are triggered by systemic lupus erythematosus combined with mixed hyperlipidemia, rather than drug-induced immune serositis. The favorable therapeutic outcomes observed in this case indicate that the dual-target therapeutic strategy with both lipid-regulating and anti-inflammatory effects may be adopted as a valuable adjuvant treatment option for patients suffering from SLE combined with chylothorax.

Several strengths and limitations of this report merit acknowledgment. The primary strength is the detailed longitudinal assessment of treatment response, with serial measurements of serum lipids and pleural effusion depth. The favorable outcome following PCSK9 inhibitor therapy suggests a potential therapeutic option for refractory chylothorax in SLE patients with hyperlipidemia. Nevertheless, several limitations should be considered. First, as a single case report, the observed therapeutic effect cannot be causally attributed to evolocumab without confirmation from larger studies. Second, the lack of thoracic duct angiography precluded precise localization of the leakage site. Third, while we hypothesize that the benefit involves dual lipid-lowering and anti-inflammatory effects, the underlying mechanisms remain speculative and warrant further investigation.

## Data Availability

The raw data supporting the conclusions of this article will be made available by the authors, without undue reservation.
